# Co-regulation of translation in protein complexes

**DOI:** 10.1186/s13062-015-0048-7

**Published:** 2015-04-25

**Authors:** Marlena Siwiak, Piotr Zielenkiewicz

**Affiliations:** Department of Bioinformatics, Institute of Biochemistry and Biophysics, Polish Academy of Sciences, Pawinskiego 5a, Warsaw, 02-106 Poland; Laboratory of Plant Molecular Biology, Faculty of Biology, Warsaw University, Pawinskiego 5a, Warsaw, 02-106 Poland

**Keywords:** Translation control, Translation regulation, Protein complex, Protein-protein interaction, Computer modeling, Protein production rate

## Abstract

**Background:**

Co-regulation of gene expression has been known for many years, and studied widely both globally and for individual genes. Nevertheless, most analyses concerned transcriptional control, which in case of physically interacting proteins and protein complex subunits may be of secondary importance. This research is the first quantitative analysis that provides global-scale evidence for translation co-regulation among associated proteins.

**Results:**

By analyzing the results of our previous quantitative model of translation, we have demonstrated that protein production rates plus several other translational parameters, such as mRNA and protein abundance, or number of produced proteins from a gene, are well concerted between stable complex subunits and party hubs. This may be energetically favorable during synthesis of complex building blocks and ensure their accurate production in time. In contrast, for connections with regulatory particles and date hubs translational co-regulation is less visible, indicating that in these cases maintenance of accurate levels of interacting particles is not necessarily beneficial.

**Conclusions:**

Similar results obtained for distantly related model organisms, *Saccharomyces cerevisiae* and *Homo sapiens*, suggest that the phenomenon of translational co-regulation applies to the variety of living organisms and concerns many complex constituents. This phenomenon was also observed among the set of functionally linked proteins from *Escherichia coli* operons. This leads to the conclusion that translational regulation of a protein should always be studied with respect to the expression of its primary interacting partners.

**Reviewers:**

This article was reviewed by Sandor Pongor and Claus Wilke.

**Electronic supplementary material:**

The online version of this article (doi:10.1186/s13062-015-0048-7) contains supplementary material, which is available to authorized users.

## Background

The majority of cellular proteins do not function in isolation, but constitute subunits of larger, stoichiometric protein complexes. In order to prevent the waste of energy and resources during the synthesis of complex building blocks, the expression of individual proteins should be precisely controlled in time and space, and with respect to other components of the complex. This may be achieved by its co-regulation at many different levels, the best studied of which is presumably transcription. Coordination of biosynthesis at this level involves many widely examined mechanisms, like formation of operons [1], proper order of genes in genomes [2], or development of transcription factors combinatorial networks [3]. It has been shown that if such co-regulation is evolutionarily conserved, the affected genes appear to participate in the same protein complexes [4-6].

In case of translational co-regulation, however, there is still much to be discovered. Although the phenomenon has been studied globally, for example, as a cell wide response to environmental stresses [7], and in some cases its detailed mechanism has been unmasked [8], little is known about translational co-regulation of complex subunits. It has recently been shown that protein complexes tend to assemble cotranslationally as one or several interacting partners are being synthesized [9]. If so, the coordination of these processes should require much control exercised at the level of translation of the individual subunits. Somewhat different, yet not mutually exclusive picture emerges from the study of dynamic complex formation during the yeast cell cycle [10], which argues that most complexes are formed by both periodically and constitutively expressed subunits. In such a case, some complex components need not be transcriptionally or translationally co-regulated in order to provide proper timing of the final complex assembly. As for the remaining subunits to which co-regulated expression may apply, the main limitation of this and other studies integrating interactomes with gene expression data (e.g. [11-13]) is the usage of mRNA abundances as the sole indicator of gene expression; this makes them again more focused on transcriptional rather than translational co-regulation.

To overcome this problem, we used the results of a computational model of translation [14] applied previously to three organisms: *Escherichia coli*, *Saccharomyces cerevisiae*, and *Homo sapiens* [15]. For each analyzed gene, the model describes its translation quantitatively in terms of several translational parameters, such as the mean time required for translation initiation in seconds, or the number of produced proteins from an mRNA during its lifetime. For the purposes of this research, we enriched the model with two additional parameters: steady-state protein abundance and average protein production rate of a gene. The extended set of parameters enabled quantitative analysis of translational co-regulation by estimating the correlation strength of parameter values among interacting proteins in three model organisms. The study was performed separately for two types of interactions: binary – detected mostly by high-throughput yeast two-hybrid system, typically enriched with regulatory and signaling inter-complex connections; and co-complex – gauged by high-throughput affinity purification followed by mass spectrometry, and reflecting the constituents of stable protein complexes [16]. As expected, the two sets differed essentially in the amount of exercised translational co-regulation. Some of the discrepancies in translational parameters values between the associated proteins were also shown, and largely explained by a detailed analysis of protein production rates among the subunits of the three yeast complexes.

Finally, we concentrated on highly connected nodes from the yeast co-complex interactions network, and compared the level of translational co-regulation between party and date hubs. As defined previously [13], party hubs represent integral proteome modules, performing some biological function, and simultaneously interact with most of their neighbors; while date hubs connect these modules and may interact with different proteins at different times and cell locations. Although the distinction between party and date hubs is not always clear cut [13], and the concept of hub dichotomy provoked many debates ([17-20]; for review, see [21]), our results revealed clear differences in co-regulation of several translational parameters between the two types of hubs.

## Results and discussion

### Translational parameters and PPIs

The following translational parameters for *E.coli*, *S.cerevisiae* and *H.sapiens* genes were downloaded from the Transimulation website [15] and summarized in Table 1: *L*, coding sequence length in codons; *x*, average number of transcripts in a cell; *g*, ribosome density in the number of ribosomes attached to a transcript per 100 codons; *w*, the absolute number of ribosomes on a transcript; *m*, estimated mean lifetime of a transcript; *I*, mean time required for translation initiation; *E*, mean time required for translation elongation; *e*, mean elongation time of one codon of a transcript; and *b*, average number of proteins produced from one molecule of transcript during its lifespan. The average number of total proteins produced from a gene was calculated as the product of *b* and *x* and marked as *B*. However, since *B* does not take into account protein degradation rates, it cannot be treated as an estimation of protein abundances. The latter were therefore obtained from several high-throughput studies [22-24] and referred to as parameter *A* (in protein molecules per cell). Additionally, a new parameter *R* was introduced, indicating the average number of proteins produced from a gene per second (for derivation, see Methods).

**Table 1:** **The summary of translational parameters calculated in the model and used in this research**

**Parameter**	**Description**
L	ORF length in codons
x	average number of transcripts per cell
g	ribosome density in number of ribosomes per 100 codons
w	number of ribosomes attached to a single transcript
m	transcript mean lifetime
I	mean translation initiation time
E	mean translation elongation time
e	mean time required for elongation of one codon of a transcript
b	number of proteins produced from a single transcript during its lifespan
B	total number of protein molecules produced from all transcripts of a gene
A	number of protein molecules per cell taken from high-throughput studies
R	average number of proteins produced from a gene per second

For each organism the sets of its binary and co-complex protein-protein interactions (PPIs) were taken from the references listed in Table 2. Due to the lack of other data for co-complex interactions in *E.coli*, we analyzed the putative complexes identified previously by clustering of physical interactions networks [25]. As the results for this set did not agree well with those obtained for co-complex interactions from other species (see below), we repeated the analysis using *E.coli* operons. Even though operon proteins need not interact physically [1,26], they are linked functionally, and thus are expected to be translationally co-regulated. This set, referred to as intra-operon proteins, may also serve as a positive control as operon genes in prokaryotes are typically transcribed as a polycistronic mRNA [1,27], and thus should have well-concerted transcript abundances *x* and lifetimes *m*. As a negative control we used a set of 3000 random interactions, generated for each species separately with the exclusion of its binary and co-complex PPIs (or intra-operon proteins). However, such random interactions are known to form networks dissimilar to those observed in biological systems. In particular, the degree distribution of random networks is often binomial (depending on how the network was constructed), while biological networks are usually scale-free [28]. To eliminate any interference stemming from this fact, we used an additional negative control – a set of interactions obtained by shuffling the nodes of the existing co-complex interactomes. Thus, the created networks have similar characteristics as their corresponding co-complex interactomes, but the connections within them are random.

**Table 2:** **The summary of PPIs sets used in the analysis**

**Organism**	**PPIs set**	**Source**	**#PPIs**	**#PPIs-h**	**#PPIs-R**
*E.coli*	binary	Rajagopala et al. [31]	2 067	1 671	427
	co-complex	Hu et al. [25]	4 515	4 515	1 219
	operon	ProOpDB, 03-04-2014 [40]	4 989	4 989	1 311
*S.cerevisiae*	binary	Yu et al. [16]	2 930	2 705	1 965
	co-complex	Yu et al. [16]	9 070	9 070	6 115
*H.sapiens*	binary	HPRD, 02-28-2014 [30]	39 240	37 039	14 395
	co-complex	HPRD, 02-28-2014 [30]	31 237	23 918	11 506

Column description: (source) the references of protein-protein interactions sets with accession dates for on-line databases; (#PPIs) number of PPIs provided by the source; (#PPIs-h) number of heterodimeric interactions in the set; and (#PPIs-R) number of heterodimeric PPIs, for which both interacting partners are attributed with the value of the protein production rate *R* – these interactions were used in the analysis.

### Correlations of translational parameters among interacting proteins

**Figure 1:**
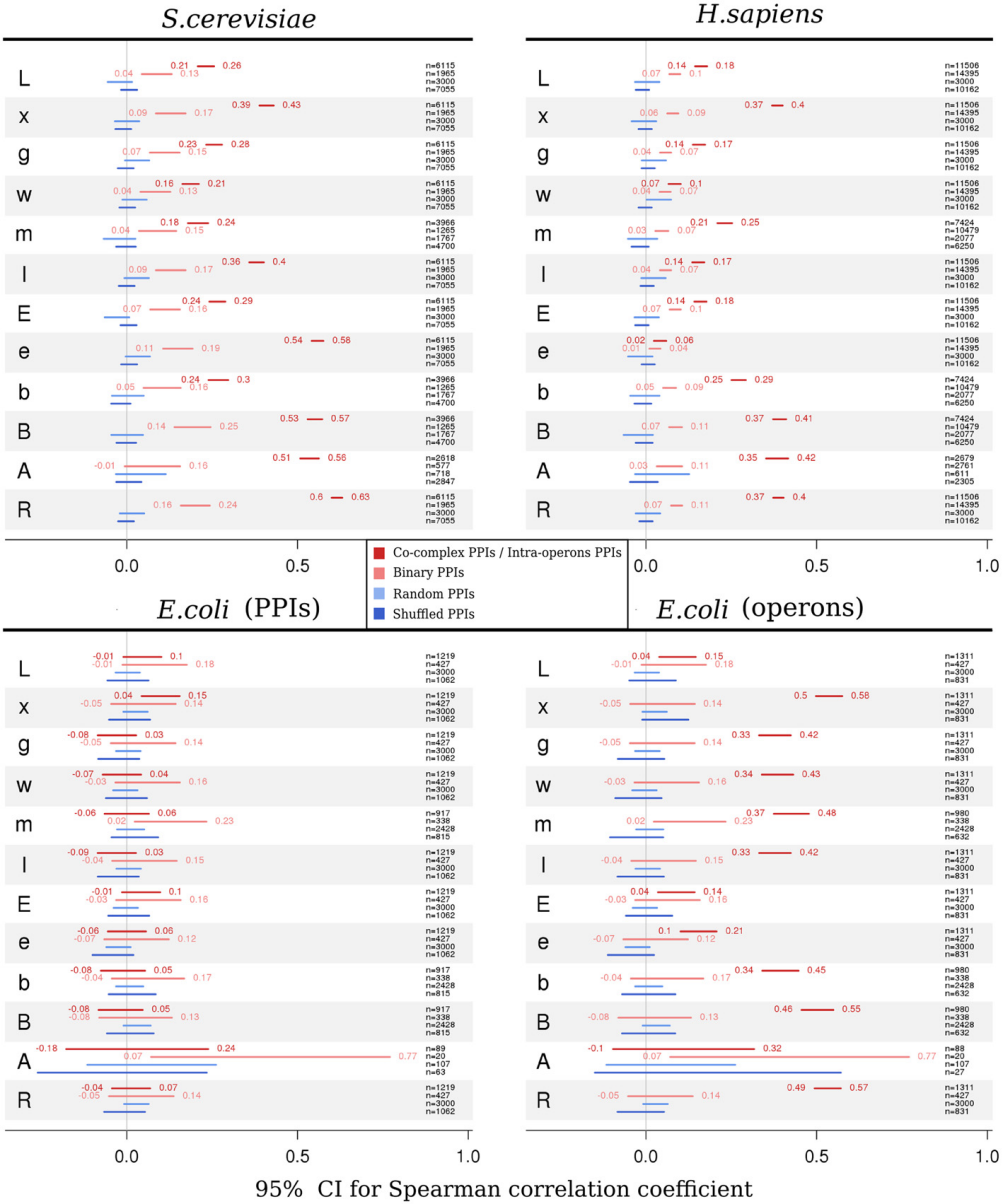
Correlation of translational parameters’ values among interacting proteins. The plots show 95% CIs for Spearman correlation coefficient calculated separately for each translational parameter between the first and second partners from a given set of PPIs. For each species, four sets of PPIs were analyzed: co-complex, binary, random, and extracted from a shuffled co-complex network. For *E.coli* the analysis was repeated using intra-operon proteins instead of co-complex PPIs (right bottom panel). For most cases, the strongest correlations are observed for co-complex PPIs (or intra-operon proteins for *E.coli*), especially for the protein production rate *R*, number of produced proteins from a gene *B*, transcript abundance *x*, and in case of yeast also mean codon elongation time *e*; *n* – sample size.

For each translational parameter we calculated from a given set of PPIs the correlation in its value between interacting partners. As the order of proteins in interacting pairs is arbitrary, in about half of the cases the value for the first partner is smaller than for the second, which results in uniform dispersion of data points below and above the 45 degree straight line (Additional file 1). The obtained correlations reflect the noisiness of this linear relationship and typically are the strongest (i.e. least noisy) for co-complex interactions. The only exceptions are the co-complex PPIs for *E.coli* as they were obtained from putative complexes determined by network clustering rather than by direct experiment. If replaced by intra-operon proteins, correlations’ strength becomes similar to that observed in yeast or humans. Correlations for binary interactions are almost always positive, but weaker than the corresponding ones for co-complex PPIs. In some cases, though, their sign cannot be determined, or the effect may be minuscule or indistinguishable from correlation sizes observed for controls. The 95% confidence intervals (CI) and sample sizes for all calculated correlations are presented in Figure 1.

Generally, the best agreement within co-complex interactions was obtained for the values of parameters *R*, *B* (also *b*), *A* and *x*. For instance, the CIs for correlation of protein production rate *R* are 0.60–0.63 in yeast, 0.37–0.40 in humans, and 0.49–0.57 in *E.coli* operons. Similar CIs, all larger than 0.37, were observed for the number of proteins produced from a gene – *B*, and transcript abundance – *x*. For protein abundance *A*, the obtained CIs are close to those of *B*, with the exception of *E.coli* for which the sample was too small (n =20) to guarantee sufficiently narrow intervals. The results for parameters related to translation time, *I*, *E* and *e*, do not allow definite conclusions. Although mean translation initiation time *I* is moderately correlated among yeast co-complex PPIs and *E.coli* operons (CI: 0.36–0.4 and 0.33–0.42, respectively), its correlation in humans is much weaker (CI: 0.14–0.17), yet still larger than in the control (e.g. CI: -0.01–0.06 for random PPIs). Analogically, although the correlation of mean codon elongation time *e* in yeast seems quite strong (CI: 0.54–0.58), the results are much weaker for *E.coli* (CI: 0.10–0.21), or close to the control for humans (CI: 0.02–0.06, while the largest absolute value of the control confidence limit is 0.05). The remaining parameters are either moderately or weakly correlated and may exhibit noticeable inter-species differences. An interesting example is provided by the case of mean transcript lifetime *m*, for which the strongest correlation was reported for *E.coli* operons (CI: 0.37–0.48), while for yeast and humans PPIs its size never exceeds 0.25. This may be explained by the fact that operon genes often share a common, polycistronic mRNA – the moment it undergoes degradation, all operon ORFs should become dysfunctional, which is reflected by similar values of *m*. Further, more detailed analyses were performed only for protein production rates *R* in yeast.

### Regulation of protein production rate global picture

**Figure 2:**
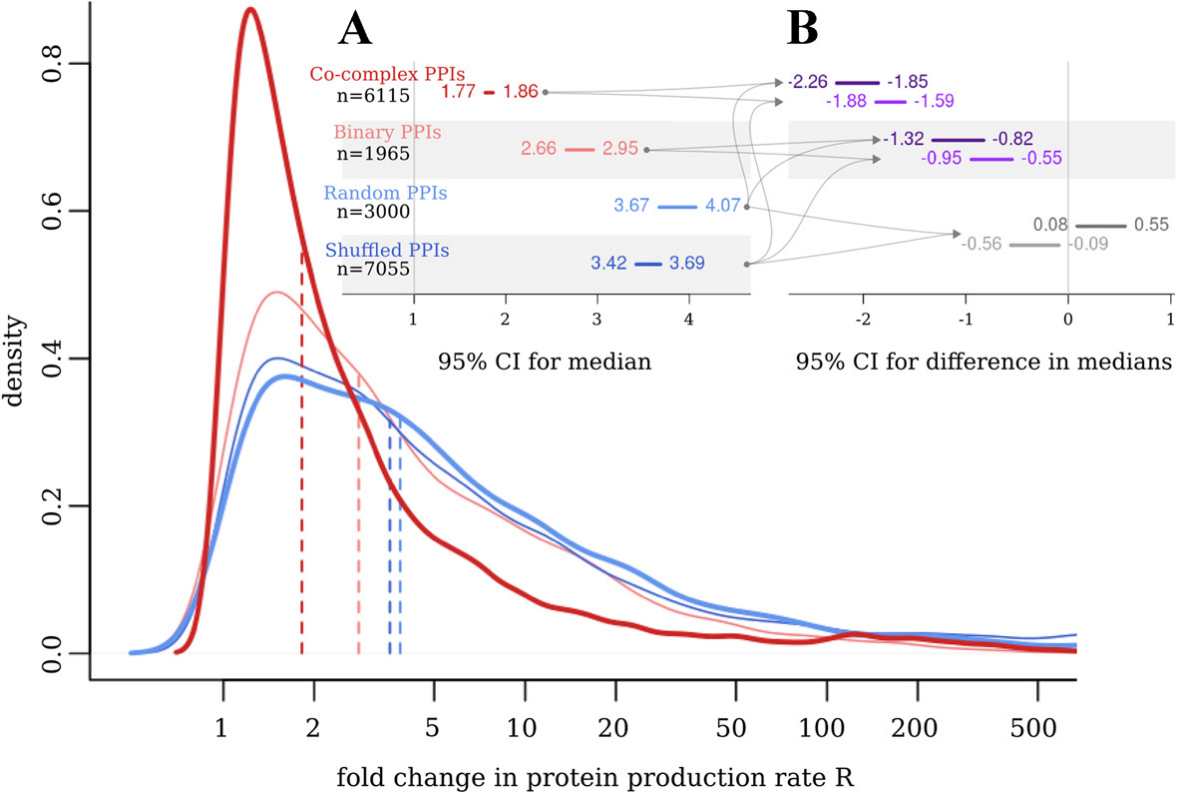
Protein production rate fold change distributions and comparison of medians. The main plot shows distributions of *R* fold change for four sets of protein pairs, as used previously. For each pair, the *R* ratio was calculated with the larger value always in the numerator; dashed lines mark median point estimators. Panel **A**: 95% CIs for distribution medians; n – sample size. Panel **B**: 95% CIs for differences in medians, with the compared medians indicated by arrows. As both control sets are equivalent, the difference in their medians was calculated twice (random PPIs median minus shuffled PPIs median, and conversely). For a typical co-complex PPI the protein production rate of one protein is 1.77–1.85 times higher than for its partner, while for random protein pairs this ratio is higher by at least 1.59 and equals about 3.40–4.00.

Our next step was to study in detail the co-regulation of translation by analyzing the differences in protein production rates *R* among interacting and random protein pairs. First, we calculated and compared the medians of *R* fold change for four sets of protein pairs previously used: co-complex PPIs, binary PPIs, random pairs and random pairs obtained from the shuffled co-complex interactome. For each analyzed protein pair the *R* ratio was calculated. To facilitate interpretation, the larger *R* value was always in the numerator, so that all obtained ratios were ≥ 1. Such a procedure enables inter-sets comparisons of modes (means or medians), which otherwise would all be close to one. It is also justified by the fact that the analyzed protein pairs are symmetrical and only the distance of *R* values between both partners is of interest, while their order is random and irrelevant. As expected, all distributions of thus computed *R* fold change are positively skewed (Figure 2), but the medians for co-complex and binary PPIs are always smaller than for random protein pairs. In particular, for a typical co-complex PPI the protein production rate *R* for one protein is 1.77–1.85 times higher than for its partner (the numbers are median 95% CI limits), while for a typical binary PPI the fold change in *R* is higher and equals 2.66–2.95. In contrast, for random and shuffled control sets, the fold change in *R* is 3.67–4.07 and 3.42–3.69, respectively (see Figure 2A). This indicates that for random protein pairs the *R* fold change is greater by at least 1.59 than for co-complex interactions, and by at least 0.55 than for binary interactions (see Figure 2B). For comparison, the differences in *R* fold change medians between two control sets range from -0.56to 0.55.

Except for medians, the distributions of *R* fold changes for PPIs and random protein pairs differ in standard deviation (sd). To quantify this difference, for each pair a logarithm of fold change in *R* was calculated, which guarantees symmetrical distribution of values around mean 0, as shown in Additional file 2 (no restriction in the choice of numerator is needed this time). For each data set the 95% CI for sd was calculated (see Additional file 2, panel A). Not surprisingly, two control sets have higher sd, plausible values of which lie between 2.17–2.31 and 2.31–2.41 for the random and shuffled data set, respectively. In contrast, sd for real PPIs log fold change distributions amount to about 1.47–1.57 and 1.72–1.86 for co-complex and binary PPIs, respectively. The difference in sd between real PPIs and random protein pairs is at least 0.64 for co-complex, and 0.35 for binary interactions (Additional file 2, panel B). For comparison, the same difference calculated between two control sets does not exceed 0.21.

### Regulation of protein production rate case study

Although the obtained correlations for the key translational parameters seem reasonably good, the agreement of parameter values for many interacting protein pairs is not always perfect. Such discrepancies may be explained by the noise in biological data or deficiencies in the computational model used to calculate values of the parameters. However, it is also possible that some of them reflect the biological functions of the proteins and their role in the complex or interactome. To illustrate this, we analyzed the details of protein production rates *R* of the components of several well-known complexes in yeast: two general transcription factors (GTFs) and a proteasome.

**Figure 3:**
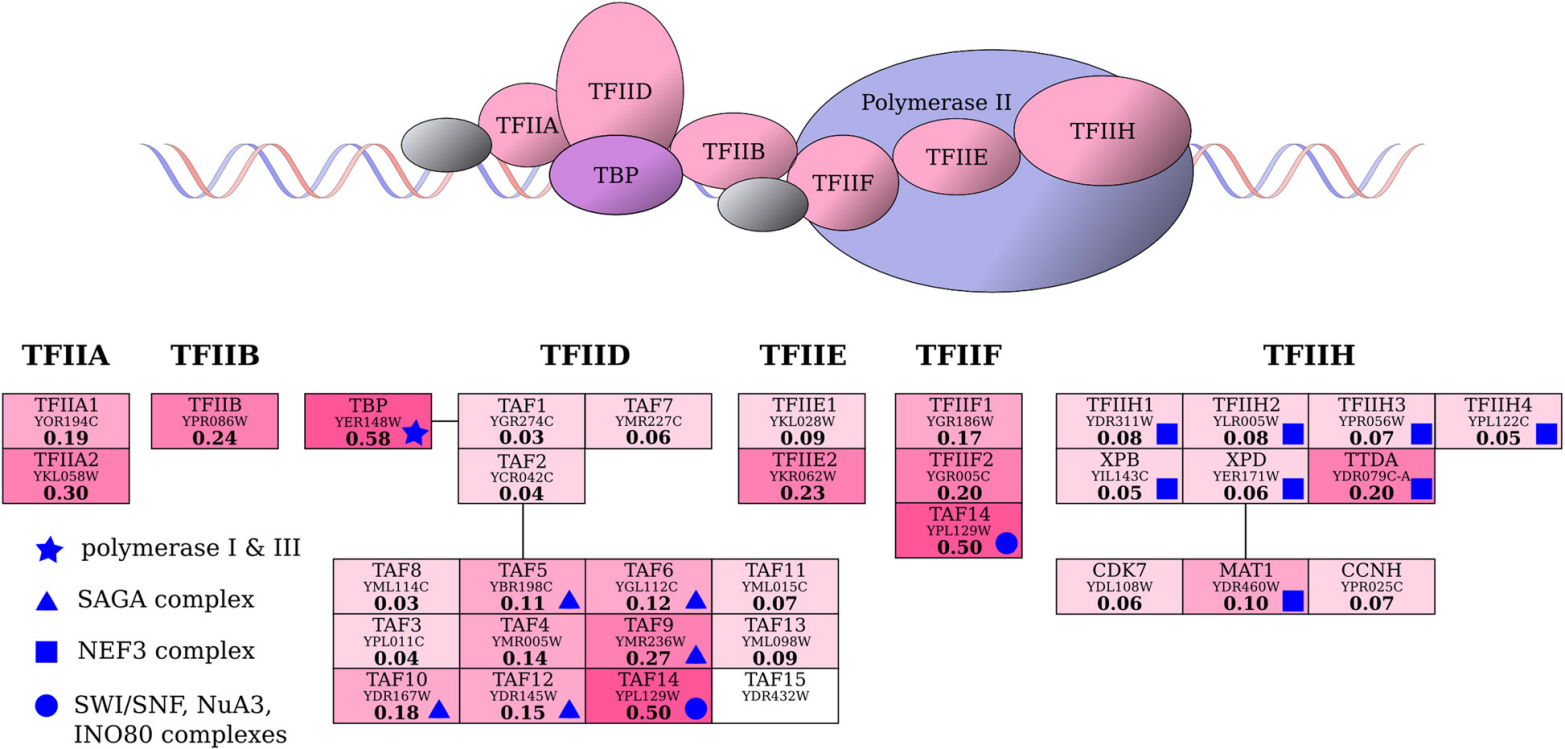
Protein production rates of general transcription factors for RNA polymerase II. Top: schematic structure of the transcription initiation complex in yeast. Bottom: composition of basal transcription factors (after KEGG [41], accessed May 2014), along with the values of the protein production rate *R* for each subunit. *R* values correspond to the color intensity. Subunits forming other complexes (according to SGD [29], accessed May 2014) are marked by blue symbols explained on the left. Protein production rates are similar for the majority of TFIID and TFIIH components. Most of the observed discrepancies in *R* may be explained by an additional biological function of a subunit in other complexes.

Transcription factors TFIIA, TFIIB, TFIID, TFIIE, TFIIF, and TFIIH constitute basal transcription factors that bind to specific sites on DNA to activate transcription, by forming an RNA polymerase II preinitiation complex. They are involved in, i.a. proper positioning of polymerase„ assembly of complex components, transcription initiation coordination and channeling the regulatory signals. The subunits of all GTFs and their production rates are presented in Figure 3. As may be seen, they do not vary much – as the *R* values of all GTFs’ subunits lie between 0.03 and 0.58, while for the entire genome between 0.0003 and 109. Nevertheless, some of the observed differences may be explained by the functions of individual proteins, as best seen for the largest complexes, TFIID and TFIIH.

For instance, TFIID consists of a TATA binding protein (TBP) and several subunits of TBP-associated factors (TAFs). Proper formation of the complex requires that all subunits are present in stoichiometric proportions at the right moment, which may be guaranteed by similar production rates of its protein components. As shown in Figure 3, such is the case for 7 out of 15 subunits of known *R*, for which it has the same order of magnitude ranging from 0.03 to 0.09. According to the Saccharomyces Genome Database [29], all these subunits are known to participate only in the TFIID complex, and thus their *R* level may be treated as the baseline for the entire complex. The remaining subunits, with the exception of TAF4, are not TFIID specific. Five of them (TAF5, TAF6, TAF9, TAF10 and TAF12), with *R* level ranging from 0.11 to 0.27, may also be found in the SAGA chromatin remodeling complex, while the TAF14 subunit of *R*=0.50 is an important component of the SWI/SFN, NuA3 and INO80 chromatin remodeling complexes. The highest *R* value is observed for TBP, which is not surprising as this is the only subunit that participates in the formation of transcription initiation complexes specific to all three types of polymerases. The second transcription factor, TFIIH, consists of ten subunits, nine of which share similar *R* values, ranging from 0.05 to 0.10, while the tenth (TTDA) has a small outstanding value of 0.20. All subunits of the TFIIH core (see Figure 3), plus MAT1, also form the nucleotide excision repair factor 3 (NEF3) complex; however, the remaining kinase CDK7 and its associated cyclin CCNH are not dissimilar in *R* values.

Another example, the proteasome, is a cylindrical protein complex which degrades unneeded or damaged proteins by proteolysis. Its core consists of two inner and two outer rings, each composed of seven individual *β* and *α* subunits, respectively. The proteolytic activity of the core is controlled by binding of the regulatory particle, built of a base and a lid of 9 subunits each, or by binding of other regulatory factors, that recognize polyubiquitin tags and initiate the degradation process. As shown in Additional file 3, protein production rates for most *α* and *β* subunits and most of the 18 subunits of the cap are all remarkably similar, and range from 0.50 to 0.76. Few subunits, *α*4, Rpt3, Rpn2, and Rpn3, show slightly lower *R* values of 0.47, 0.42, 0.35, and 0.40, respectively, whereas subunits *α*5, Rpn13, and Rpn15 show elevated rates of respectively 1.04, 0.85, and 1.49. In case of the Rpn15 subunit, this may be explained by the fact that it also forms a TREX-2 complex involved in mRNA export from the nucleus. In contrast, an important proteasome activator PA200, which should be needed in smaller amounts than the proteasome itself, has a one order of magnitude lower protein production rate (*R*=0.05).

### Translation regulation in party and date hubs

**Figure 4:**
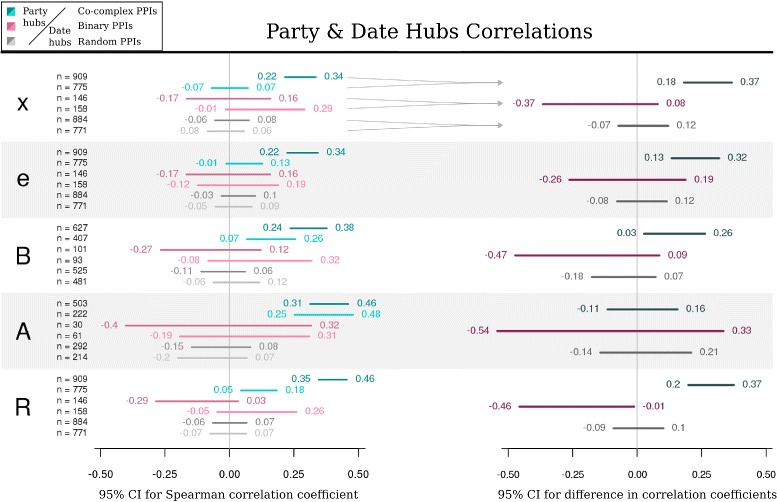
Correlations of translational parameters’ values within interactions of party and date hubs in yeast. Left: 95% CIs for Spearman correlation coefficient calculated for translational parameters *x*, *e*, *B*, *A*, and *R* between the first and second partners of the given set of PPIs. The PPIs sets were prepared by extracting all interactions for party (dark colors) and date hubs (light colors) from co-complex PPIs network (green), and binary PPIs network (magenta). Random PPIs (gray) were prepared as described in Methods; n indicates the number of protein pairs in each subset. Right: 95% CIs for difference in correlation coefficients between party and date hubs; for each translational parameter the difference was calculated separately for each type of PPIs. For all parameters, except protein abundance *A*, correlations are the strongest for co-complex PPIs of party hubs. The results for the remaining parameters are shown in Additional file 4.

We also calculated and compared the agreement in values of translational parameters between party/date hubs and their interacting partners. For each of the 91 date hubs and 108 party hubs identified previously in yeast [13], we extracted its PPIs from the co-complex and binary interactomes. As a control we used the sets of random interactions of party and date hubs, prepared as described in Methods. Next, for each translational parameter its agreement within protein pairs was calculated as previously, i.e. by calculating 95% CIs for the Spearman correlation coefficient. The results for five parameters, *x*, *e*, *B*, *A* and *R*, which had a correlation of at least 0.39 in the general analysis of co-complex associations, are presented in the left panel of Figure 4, while the right panel shows 95% CIs for correlation differences between party and date hubs for co-complex PPIs (i), for binary PPIs (ii), and for random PPIs (iii).

In case of four parameters, *e*, *x*, *R* and *B*, party hubs show stronger agreement than date hubs, but only within co-complex interactions. The largest difference is observed for the protein production rate *R* and transcript abundance *x*, which is within 0.2–0.37 and 0.18–0.37, respectively. For comparison, the correlation differences between party and date hubs random PPIs are not larger than 0.1 and 0.12 for *R* and *x*, respectively; moreover their signs cannot be determined. For the mean codon elongation time *e* this difference may be a bit smaller, as its CI for co-complex PPIs ranges from 0.13 to 0.32, and is only a little above the upper confidence limit of the control correlation difference. Furthermore, for the number of produced proteins *B* party-date correlation difference lies within 0.03–0.26 (for comparison, the control CIis -0.18–0.07), and we cannot exclude the possibility that it is negligible. For protein abundance *A* both party and date hubs exhibit moderate, yet very similar correlations, within 0.31–0.46 and 0.25–0.48, respectively; hence the sign of the difference between them cannot be determined (CI: -0.11–0.16).

In case of binary interactions, the sample sizes are several times smaller, which leads to wider CIs and may hide underlying effects. For instance, for all parameters the observed correlations between hubs and their partners cannot even be claimed as positive or negative. Nevertheless, taking into account their upper CI limits, as well as the results presented in Figure 1, one should not expect effects larger than for co-complex PPIs. Also the results for the remaining translational parameters *L*, *g*, *w*, *m*, *I*, *E*, and *b* are not informative – the sign of the obtained correlation difference cannot be determined, or, in case of *w*, its size is very difficult to interpret due to statistical uncertainties (seeAdditional file 4).

## Conclusions

In this paper we have analyzed the results of our previous quantitative model of translation [14] in the context of protein-protein interactions networks and revealed that among interacting proteins translation is co-regulated at many different levels. The values of several translational parameters, for instance, mRNA and protein abundance, number of produced proteins from a gene, or, newly introduced, protein production rate, agree well between interacting partners and consequently for both human and yeast. This was further confirmed by the analysis of the protein production rate in yeast co-complex PPIs, which revealed that its fold change between interacting proteins is typically smaller by at least 1.59 than in case of random protein pairs (Figure 2), while standard deviation of its log fold change distribution is smaller by at least 0.64 (Additional file 2).

The agreement between the values of translational parameters is visible even despite the fact that most proteins interact with more than one partner, which makes translation co-regulation a multidimensional process. In such a case, two-dimensional correlations may only roughly outline the existing dependencies, and are prone to “outliers” such as regulatory subunits, whose translational regulation may be distinct from that of the remaining components of a complex. This phenomenon was depicted by the example of general transcription factors (Figure 3), and to a lesser extent – proteasome (Additional file 3), where many subunits produced in excess in relation to the complex baseline were additionally involved in formation of other functional modules. Moreover, one must not forget that the underlying translational model is only theoretical, and that the quality of its outcome strongly depends on the quality of input data. In consequence, some of the observed discrepancies may reflect estimation errors caused by biological noise or model drawbacks. Finally, although the strength of the obtained correlations is sufficient for some general conclusions, it does not seem large enough to enable more sophisticated analysis, such as relevant prediction of existing PPIs on the basis of translational parametersvalues.

Another conclusion of this research concerns the nature of the used protein-protein interactions data sets. For all performed analyses, the best results were obtained for co-complex interactions, while correlations for binary PPIs were typically much weaker, and in many cases indistinguishable from the results for the control sets of random interactions. Also the analysis of protein production rate (fold change) revealed that its distribution for binary PPIs resembles more closely the distributions for random PPIs, while the distribution for co-complex PPIs has visibly smaller median and standard deviation (for logged values). All this may be caused by lower quality of binary PPIs. However, the opposite was shown for at least two yeast sets [16]. Also, it should be stressed that many low-affinity PPIs may be undetectable by methods such as yeast two-hybrid system, causing their under-representation in the binary sets that may bias the outcome. Nevertheless, another explanation is also possible – that for transient connections with regulatory and signaling proteins, maintenance of adequate proportions of synthesized complex subunits, and thus their translational co-regulation, is not always economically beneficial. Future research will aim to check whether such binary interactions may be assigned to periodically expressed complex subunits [10], mentioned in theIntroduction.

Instead, the limited quality may be an issue in case of co-complex PPIs detected by non-experimental methods, as for *E.coli* co-complex PPIs. In particular, within this set we were unable to determine the correlation sign for any of the studied parameters except mRNA abundance *x*, but even in this case, the correlation was much weaker than the ones observed for yeast and humans (Figure 1). Only after replacing the *E.coli* co-complex set with intra-operon associations, the obtained results became coherent for all the studied species. Another advantage of using operons is that they are transcriptionally co-regulated, and even though their definition does not require a polycistronic mRNA, it is often so in practice [1,27]. If so, some parameters, such as transcript abundance *x*, or mean mRNA lifetime *m* should have almost identical values for all genes from a given operon, resulting in size of correlations close to one. The fact that the observed correlations are not larger than 0.58 (for protein abundance *A* the upper limit is even 0.77, but the correlation CI is burdened with a high degree of uncertainty) indicates that data used to gauge translational parameters are still very noisy, or, less likely, the methods used to detect operons need to improve. On the other hand, however, operon analysis may serve as a positive control defining the limit of detectable co-regulation level of interacting proteins, given all methodological constraints. Therefore, obtaining correlations of similar sizes for many parameters within co-complex PPIs in yeast and humans suggests that we have approached the maximum detectable effect, which further confirms the quality of our computational model of translation and the examined co-complexPPIs.

Moreover, the analysis of translational co-regulation among yeast hubs showed that for party hubs, which represent integral elements within distinct proteome modules, the mRNA abundance *x*, protein production rate *R*, mean codon elongation time *e*, and possibly the number of produced proteins from a gene *B* are more concerted between the hub and its partners than for date hubs, which act as signal mediators between these modules (Figure 4). In contrast, protein abundance *A* may besimilarly well concerted for both types of hubs, while for the remaining translational parameters the effect seems either very weak or could not be detected in this analysis. It should be noted, however, that in case of transcript abundance *x* the results are not surprising, as the distinction between party and date hubs was made according to the values of correlation coefficients between the hub and each of its neighbors for mRNA expression [13]. Inparticular, hubs with correlation below the cutoff of 0.5 were named date hubs, and above – party hubs. In such a case, some proteins interacting with party hubs according to our PPIs sets should have more similar values of parameter *x*, resulting in larger correlations. Our findings meet these expectations, showing additionally that expression co-regulation among party hubs and their partners is not limited to the mRNA transcription level. This may prove useful in detecting new date hubs, e.g. in pathogens, as they are considered the best targets for therapeutic intervention [13].

Summing up, this work provides global-scale evidence for translation co-regulation among interacting proteins. It is best visible while studying protein production rates among components of stable complexes, indicating that its main purpose is to prevent waste of resources during synthesis of building blocks of stoichiometric complexes and guarantee their on time production. Similar results obtained for distantly related species suggest that this phenomenon applies to the variety of living organisms and concerns physically interacting as well as functionally linked proteins, as shown by the analysis of intra-operons proteins in *E.coli*. Its prevalence and the fact that proteins usually do not operate in isolation lead to the conclusion that regulation of a protein translation should always be studied with respect to expression of its primaryinteracting partners.

## Methods

### Derivation of protein production rate

Translational parameters for 1738 *E.coli*, 4470 *S.cerevisiae*, and 7497 *H.sapiens* genes were downloaded from the Transimulation website [15]. For their derivation, see the quantitative model of translation [14]. Additionally, a new translational parameter – protein production rate – was calculated for each gene on the basis of the existing parameters. Under the assumption of a transcriptional steady state, i.e. the same number of matured mRNAs appearing in the cytoplasm as being degraded, the protein production rate *R* for the gene *i* equals:

(1) $$ R_{i} = x_{i} \cdot \frac{b_{i}}{m_{i}},  $$

where *x*_*i*_ stands for the number of mature transcripts of gene *i* present in the cell, *b*_*i*_ is the number of proteins produced from one such transcript during its lifetime, and *m*_*i*_ is its mean lifetime in seconds. Thus, obtained *R*_*i*_ is expressed as the number of protein molecules produced per second by the gene’s mRNAs present in the cell at that time. Due to the input data limitations of the underlying translational model, the values of *b* and *m* were provided only for the fraction of analyzed genes. To solve this problem, we substituted *b* with the quotient of *m* and *I* – the mean translation initiation time of a given mRNA, as explained in the core model publication [14]. In consequence, *R*_*i*_ may be calculatedsimply as:

(2) $$ R_{i} = \frac{x_{i} \cdot m_{i}}{m_{i} \cdot I_{i}} = \frac{x_{i}}{I_{i}}.  $$

### Protein-protein interactions

The sets of binary and co-complex interactions for all three species, along with their references, are summarized in Table 2. Only if the interaction was heterodimeric and both proteins had attributed translational parameters values, it was retained for further analysis. For yeast, the “Y2H-union” and “Combined-AP/MS” sets were used as binary and co-complex PPIs, respectively [16]. In case of humans, the HPRD database [30] classifies all experimentally derived PPIs into binary, i.e. direct interactions between two proteins, or complex, representing interactions of unknown topology with more than two partners. In case of *E.coli*, binary PPIs were gauged by yeast two-hybrid screening [31], while multiprotein complexes were predicted by clustering of protein physical interactions network [25]. For the purpose of this research, we obtained direct co-complex associations within *E.coli* and human sets by forming all possible, non-redundant pairs of proteins from each distinguished complex. An additional set of *E.coli* protein pairs was prepared based on the operon genes determined elsewhere, as shown in Table 2. In this case, for each reported operon we generated all possible protein pairs between the protein products of its genes and refer to them as intra-operon proteins. If necessary, protein names were transformed with the help of the Uniprot on-line tools [32].

For the main analysis, random PPIs sets were generated for each species separately by sampling with replacement of protein pairs from the set of proteins of known translational parameters, with exclusion of existing (binary, co-complex or intra-operon), redundant and homodimeric interactions. The sampling was repeated until 3000 random PPIs were obtained. The shuffled-interactome PPIs (referred to as “shuffled PPIs”) were generated by shuffling protein locations in the co-complex interactome of each species, followed by the extraction of all protein pairs from the created network. The shuffling procedure yielded the same number of random interactions as those present in the corresponding co-complex interactomes. For the *E.coli* operon analysis, the shuffled PPIs were generated by shuffling locations of genes in the operons, followed by the extraction of all possible pairs of their protein products from each of the created falseoperons.

### Party and date hubs

The lists of 108 party and 91 date hubs of *S.cerevisiae* were taken from ref. [13]. For each hub its interactions were extracted from the binary and co-complex PPIs sets prepared previously for yeast. As a result, we obtained 199 binary and 1024 co-complex interactions for party hubs, and 187 binary and 891 co-complex interactions for date hubs. Control sets of random PPIs were generated separately for party and date hubs. For each hub of known translational parameters, *n* protein partners were sampled without replacement from the set of proteins of known translational parameters. The value of *n* was set according to the number of real neighbors of known translational parameters for this hub in the co-complex network. Thus, we obtained 884 and 771 random PPIs for party and date hubs controls, respectively.

### Statistical analysis

All correlations reported in our analysis are the nonparametric Spearman correlations. The 95% confidence intervals (CIs) for correlation coefficients were calculated using standard tools from the R environment. Correlation differences CIs were calculated with the help of the cocor.indep.groups function from the cocor R package [33], with the zou2007 method applied [34].

95% CIs for medians and medians’ differences of the *R* fold change, as well as 95% CIs for standard deviations and standard deviations’ differences of log fold *R* change were calculated with the help of the simpleboot R package [35]. The CI limits were gauged by both normal approximation and a percentile method – both calculations variants returned almost identical results, and thus only normally approximated CIs were shown. Bootstrap samples were 2000 for all analyses.

## Reviewers’ comments

### Reviewer report 1: Prof. Sandor Pongor, International Centre for Genetic Engineering and Biotechnology (ICGEB), Italy

The study of Siwiak and Zielenkiewicz examines the co-regulation of translation rates of interacting proteins, using analysis of correlations of several translational parameters assigned to proteins in the interaction network. These parameters are derived from a computational model of translation described in the authors’ previous works and further developed in this paper. The main conclusion of this research is that members of stable complex subunits and party hubs tend to be translationally co-regulated, which is not the case for interactions with regulatory proteins and date hubs. The authors suggest that such regulation may be “energetically favorable” and facilitate complex formation.

The presented article regards important an interesting, yet very weakly understood natural phenomenon. The authors present convincing analysis based on well-designed tests performed on carefully chosen protein-protein interaction data. The authors have chosen to report the correlations as confidence intervals instead of just p-values, offering readers much more reliable and comprehensible results. Generally, the text is well written and suitable for publication in BiologyDirect.

Minor things:

1) Weak correlations for binary PPIs sets, received mainly from Y2H experiments, may be caused by limitation of this experimental methodology in detection of low-affinity protein-protein interactions (requiring higher concentrations of substrate subunits). Such limitations should be mentioned in the discussion.

2) Page 1: Abstract, Conclusions. It is not clear to me if the results obtained for *E.coli* are indeed similar to those for other two organisms, as the set used for *E.coli* (“intra-operon PPIs”) was treated as a “positive control”. Besides, the name of this set is confusing. Although authors stressed that this set does not need to include physically interacting proteins, the acronym “PPI” suggests it.

3) Page 5: What does “modes” in “comparisons of modes” refer to?

4) Page 9-10: Inconsistent use of the index “i”, e.g. “i gene’s mRNAs”.

5) Page 13: Line 2, “transcritption” instead of “transcription”.

6) General remark: A table of parameters (including abbreviations) used for calculations would be very helpful.

*Authors’ response: We agree with the prof. Sandor Pongor comments and have corrected the manuscript accordingly. As for the second remark, indeed, the intra-operon set is different, as many proteins produced from one operon do not interact physically, which we mentioned in Results before. They are, however, linked functionally and may also be translationally co-regulated. Although the abstract length is limited and does not allow for more detailed explanations, we decided to signal this distinction at least. Also, all instances of the misleading term “intra-operon PPIs” were substituted with “intra-operon proteins”.*

### Reviewer report 2: Dr Claus Wilke, The University of Texas at Austin, United States of America

The paper attempts to study the extent to which interacting proteins are co-translationally regulated. This is an interesting question; however, I’m not entirely convinced that the available data are of sufficiently high quality. Therefore, I’m not sure how reliable the conclusions are that are presented here.

First, all the translational data basically stems from a fit of a model, not from individual measurements. By the authors’ own words from their 2010 paper, their model yielded only “reasonably good correlations” with available data. I’m not sure how much we can trust such a model when we then want to use it to make downstreaminferences.

*Authors’ response: We share these doubts with dr Claus Wilke and are aware of the limitations of this research and the former model, which was stressed in Conclusions. However, we are also aware that the quality of the model outcome cannot be larger than the quality of its input on which we have no influence. We try to choose the most reliable data but even sets from highly published papers raise some quality concerns (for instance, lack of measurements precision estimates). In our previous paper [*15*], we have demonstrated that for some parameters the predictions of our model show similar agreement with experimental studies as the experimental studies among themselves. But these experimental studies can explain even as little as 12% of their counterpart variance. Replicability of high-throughput experimental measurements was also discussed by others, e.g. [*36*]. Based on such observations, we believe that “reasonably good correlations” is the best we can achieve in this field with the current technology. Despite these limitations, some signal is still visible, especially in comparison with negative and positive controls. It is hard to believe that it is entirely caused by the noise. Nevertheless, taking into account its strength and the complexity of the analyzed matter, we try to be very cautious with our conclusions and discuss our concerns honestly in the paper.*

Second, several of the protein-protein interaction data sets are also dubious. In particular, yeast two-hybrid measurements contain such a large amount of false positives that they are basically useless. Further, the authors admit that the *E.coli* data are not very good but do the analysis anyway. Why not just leave out *E.coli* if the data availability is poor?

*Authors’ response: For the most extensively used Y2H set (binary PPIs for yeast) the quality seems at least acceptable and comparable to the quality of data from affinity purification followed by mass spectrometry (see the reference paper [*16*]). Besides, it is not always obvious which data sets are useless or of poor quality before one tries to perform an analysis on them (as in the case of E.coli co-complex set). We believe that after the analysis is finished all such disappointing results should be reported with an appropriate commentary. Note that none of the papers on PPIs we cite was retracted so far. Other researchers may want to use them in their own research, unless they find similar reports to ours. Showing how badly these data perform in comparison with other sets may save others a lot of time and money. Sweeping these facts under the rug leads instead to false beliefs that high-throughput experimental measurements (e.g. on PPIs, or translational parameters discussed above) are more reliable than they really are, and may provoke excessive expectations in relation to the results that are realistically possible to obtain.*

Overall, when reading this paper, I felt like I just had just been sent back in time by about a decade. For example, the authors refer to a 2006 paper as “recent”. Moreover, the idea of date and party hubs, in particular, was highly fashionable around 2005-2007 but most analyses since have shown that that distinction is likely not very meaningful. For example, after about 5 minutes of googling, I came across this paper: Agarwal et al., Plos Comput Biol, 2010, doi:10.1371/journal.pcbi.1000817, which looks highly relevant but is not cited by theauthors.

*Authors’ response: Indeed, the word “recently” may not be relevant here and we have corrected it. Nevertheless, we still find the mentioned paper worth citing, as it was presumably the first critique of the party and date hubs concept published in the times when the idea was still highly fashionable. In that case, other criticizing papers published since 2007 are newer, but not that novel. Although the debate about party and date hubs was signaled in the introduction before, we decided to supplement it with some up-to-date references, as suggested by dr Claus Wilke. Additionally, some of them show that hub dichotomy is still an open problem(e.g. [*21*]).*

Minor comments: “In particular, the degree distribution of random networks is binomial”. That depends on how the random networks were constructed.

*Authors’ response: We agree and have corrected the text accordingly.*

I don’t understand this sentence on p. 4: “In some cases, though, their sign cannot be determine”. How can we not determine the sign of a correlation coefficient? Once the coefficient is calculated, we can look at it and see the sign. If the authors want to express that the coefficient is not significantly different from zero, then they need to say that.

*Authors’ response: Typically, researchers report only point estimates of correlation coefficients along with their p-values. Such an approach has many drawbacks [*37*-*39*], thus we adopt thinking in terms of confidence intervals throughout the entire paper. Here, the obtained confidence interval for correlation coefficient contained both negative and positive values (e.g. [-0.1, 0.15]), thus the true value of the coefficient (i.e. for the entire population, not just sample) lies anywhere within this interval with 95% confidence. This value may be negative or positive, but we were not able to determine it more precisely in our research. Writing instead that “correlation is not significantly different from zero” is very often interpreted as “correlation equals zero” or “there is no correlation”, which is not true, as further research on larger samples may show that its CI lies entirely, e.g., above zero. To avoid these and other misunderstandings, we concentrate on interval estimates and magnitude of the observed effects, which we find more favorable than the popular statistical significance approach.*

## Additional files

Additional file 1
**Figure S1.** Scatter plots showing agreement in protein production rates among interacting proteins.

Additional file 2
**Figure S2.** Protein production rate log fold change distributions and standard deviations comparisons.

Additional file 3
**Figure S3.** Protein production rates of proteasome subunits.

Additional file 4
**Figure S4.** Correlations of translational parameters’ values within interactions of party and date hubs in yeast.

## Abbreviations

PPI: Protein-protein interaction
CI: Confidence interval
TF: Transcription factor
GTF: General transcription factor
TBP: TATA binding protein
TAF: TBP-associated factor
