# Supplementary material for: Co-regulation of translation in protein complexes
Source: Biol Direct. 2015 Apr 25;10:18. doi: 10.1186/s13062-015-0048-7 (PMC4409705; doi:10.1186/s13062-015-0048-7)
Supplement: Additional file 1 — Figure S1. Scatter plots showing agreement in protein production rates among interacting proteins. [file 13062_2015_48_MOESM1_ESM.pdf]

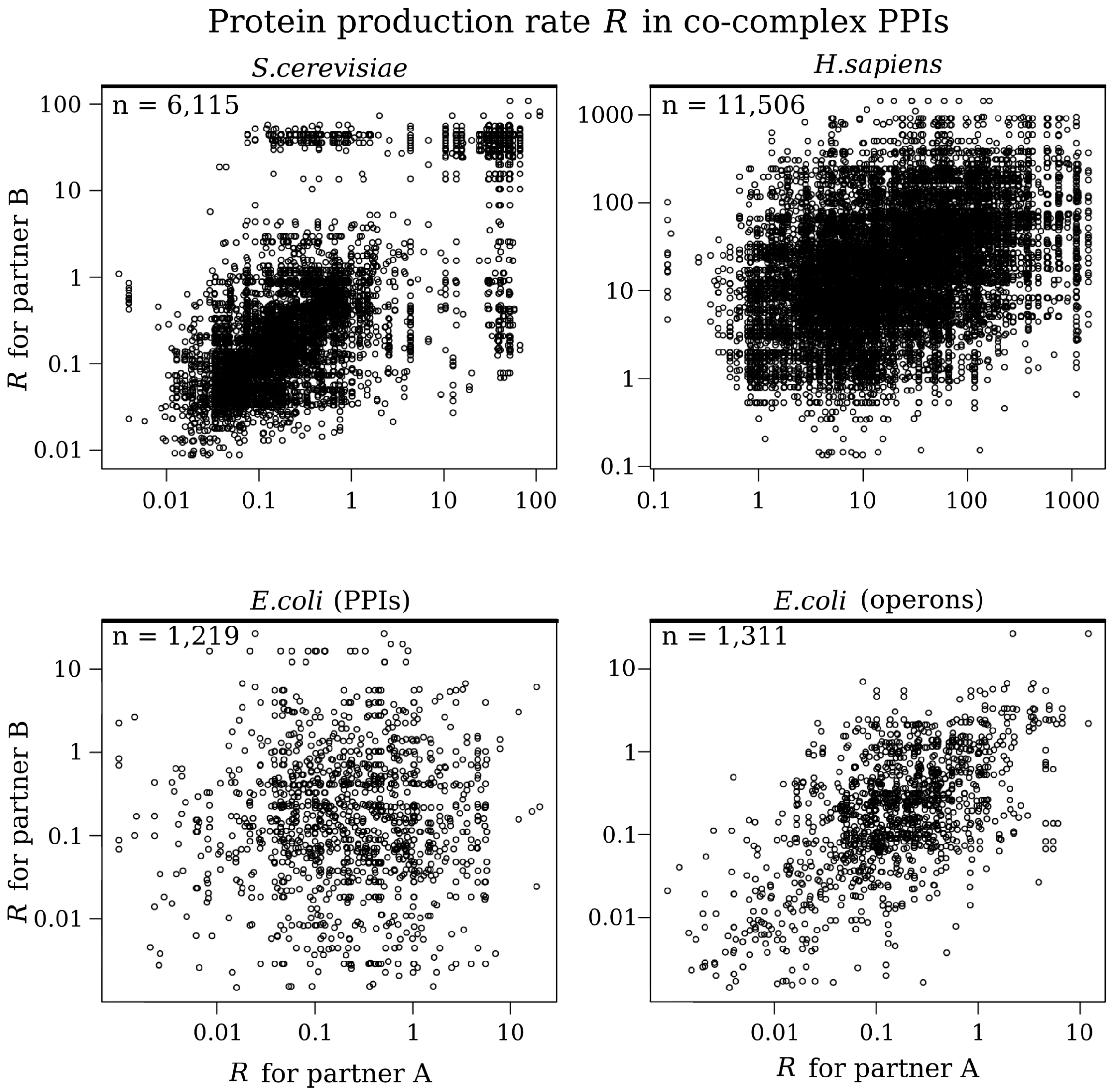

Figure S1: **Agreement in protein production rates among interacting proteins.** Scatter plots show protein production rates R for interacting proteins from co-complex PPIs or intra-operon proteins (*E.coli*) networks. X and Y coordinates of each point correspond to R values for two interacting partners. As the order of proteins in the associated pair is arbitrary, the parameter value for the first partner is smaller than for the second one in about half of the cases, which results in a uniform dispersion of data points below and above the 45 degree straight line; n – number of analyzed protein pairs.
