# Supplementary material for: Co-regulation of translation in protein complexes
Source: Biol Direct. 2015 Apr 25;10:18. doi: 10.1186/s13062-015-0048-7 (PMC4409705; doi:10.1186/s13062-015-0048-7)
Supplement: Additional file 2 — Figure S2. Protein production rate log fold change distributions and standard deviations comparisons. [file 13062_2015_48_MOESM2_ESM.pdf]

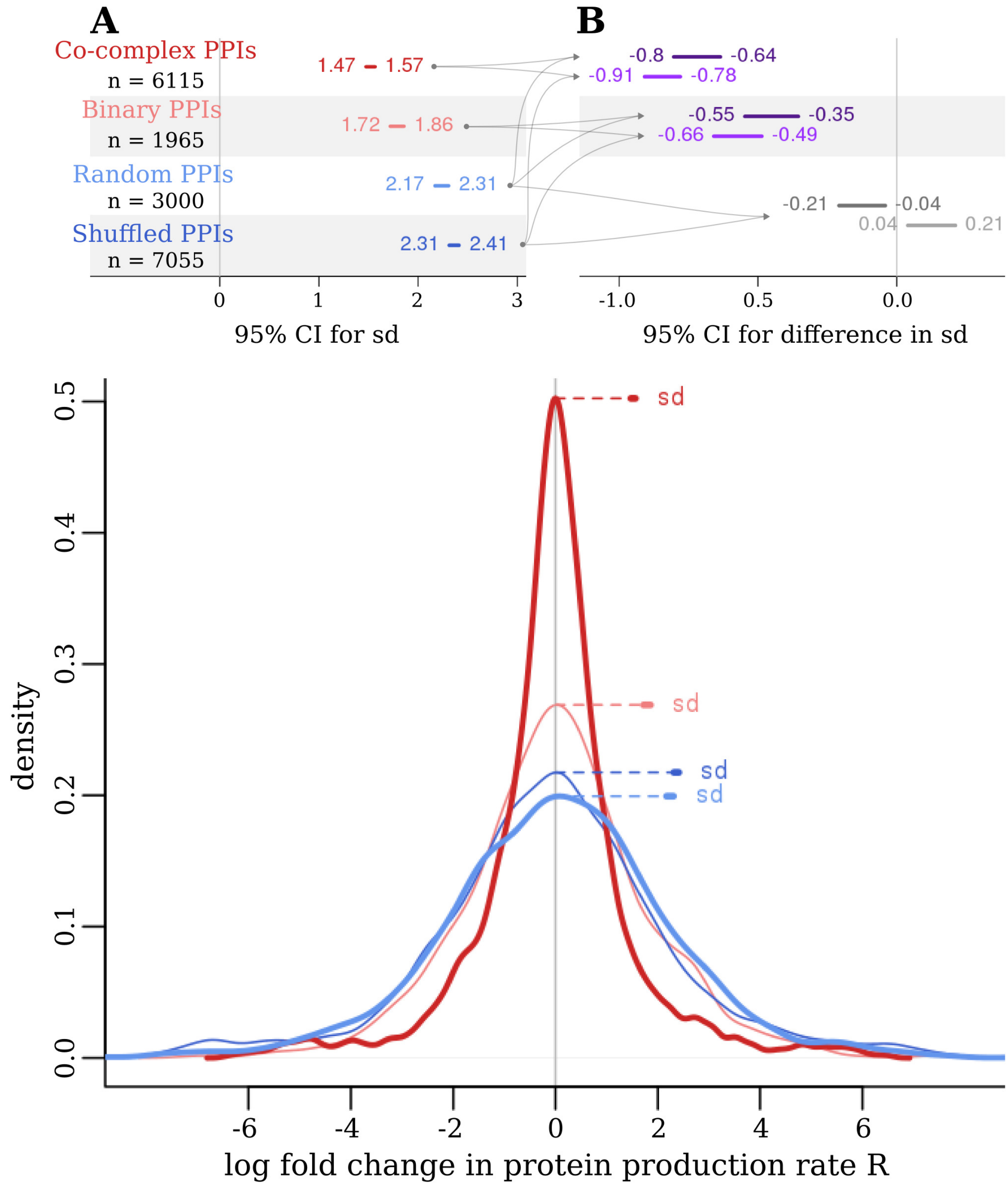

Figure S2: **Protein production rate log fold change distributions and standard deviations comparisons.** The main plot shows distributions of R log fold change for four sets of protein pairs, as used previously. The length of dashed lines indicates the lower confidence limit of the corresponding 95% CI for standard deviation (sd); the length of the solid horizontal lines stands for the CI's width. Panel A: 95% CI limits for sd; n – sample size. Panel B: 95% CI limits for sd differences, the compared standard deviations are indicated by arrows. For comparison between two control sets, sd difference was calculated twice (random PPIs sd minus shuffled PPIs sd, and conversely), as both control sets are equivalent. Two control sets have higher standard deviations of R log fold change distributions than the co-complex set (by at least 0.64) and binary set (by at least 0.35), while the sd difference between them does not exceed 0.21.
