# Supplementary material for: Co-regulation of translation in protein complexes
Source: Biol Direct. 2015 Apr 25;10:18. doi: 10.1186/s13062-015-0048-7 (PMC4409705; doi:10.1186/s13062-015-0048-7)
Supplement: Additional file 3 — Figure S3. Protein production rates of proteasome subunits. [file 13062_2015_48_MOESM3_ESM.pdf]

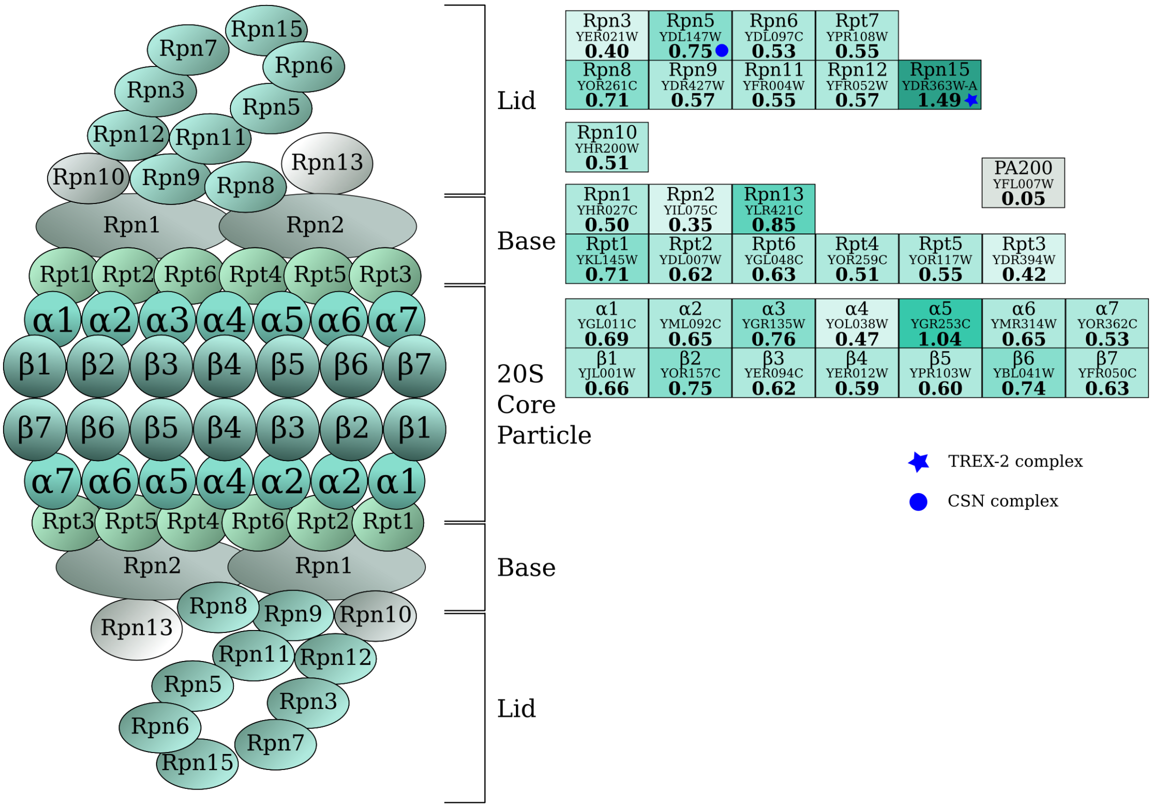

Figure S3: **Protein production rates of proteasome subunits.** Left: schematic structure of the proteasome complex in yeast. Right: composition of the proteasome 20S core particle, base and lid (after Kyoto Encyclopedia of Genes and Genomes [40], accessed May 2014), along with the values of the protein production rate R for each subunit. R values correspond to the color intensity. Subunits forming other complexes (according to Saccharomyces Genome Database [29], accessed May 2014) are marked by blue symbols, whose meaning is explained on the right. Protein production rates are similar for the majority of proteasome components. The observed discrepancies in R for Rpn15 and Rpn5 may be explained by additional biological functions of these subunits in other complexes.
