# Supplementary material for: Co-regulation of translation in protein complexes
Source: Biol Direct. 2015 Apr 25;10:18. doi: 10.1186/s13062-015-0048-7 (PMC4409705; doi:10.1186/s13062-015-0048-7)
Supplement: Additional file 4 — Figure S4. Correlations of translational parameters’ values within interactions of party and date hubs in yeast. [file 13062_2015_48_MOESM4_ESM.pdf]

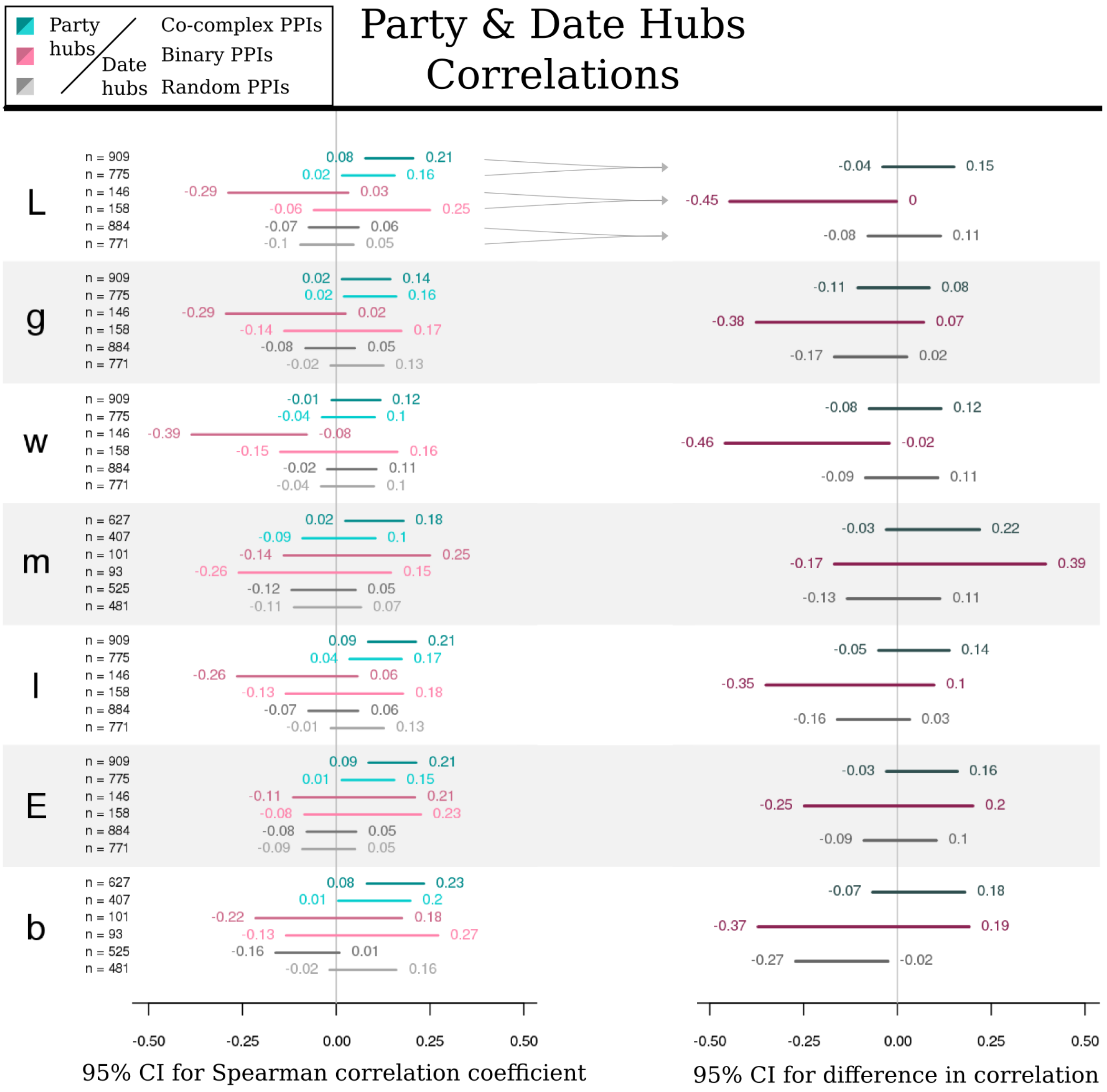


Figure S4: **Correlations of translational parameters' values within interactions of party and date hubs in yeast.** Left: 95% CIs for Spearman correlation coefficient calculated for translational parameters $L$, $g$, $w$, $m$, $I$, $E$, and $b$ between the first and second partners for each set of PPIs. The PPIs sets were prepared by extracting all interactions for party (dark colors) and date hubs (light colors) from co-complex PPIs network (green), and binary PPIs network (magenta). Random PPIs (gray) were prepared as described in Methods; n indicates the number of protein pairs in each subset. Right: 95% CIs for difference in correlation coefficients between party and date hubs; for each translational parameter the difference was calculated separately for each type of PPIs. For all analyzed parameters except $w$ the sign of the correlation difference between party and date hubs (for both co-complex and binary PPIs) cannot be determined. The results for the remaining translational parameters are shown in Fig. 4, main text.
